# Inter-professional collaboration and associated factors among nurses and physicians in specialized public hospitals, the northwest, Ethiopia: mixed method multi-centered cross-sectional study

**DOI:** 10.1186/s12913-023-09200-5

**Published:** 2023-03-27

**Authors:** Tadele Degu, Eden Amsalu, Awoke Kebede, Ousman Adal

**Affiliations:** 1 Department of pediatrics, Bahir Dar University College of medicine and health sciences, Bahir Dar, Ethiopia; 2 Department of emergency, Bahir Dar University College of medicine and health sciences, Bahir Dar, Ethiopia

**Keywords:** Nurse, Physician, Associated factors, Collaboration

## Abstract

**Objective:**

The study aimed to investigate inter-professional collaboration and associated factors among nurses and physicians working in referral and teaching hospitals in the Northwest and Ethiopia in 2022.

**Method:**

This study used a concurrent (quantitative cross-sectional and phenomenological qualitative) design from September to October 2022. A structured, self-administered nurse-physician collaborative scale questionnaire was used to collect quantitative data from 279 nurses and 87 physicians. A simple random sampling technique was used to select participants. The magnitude of the association was measured using the odds ratio at a 95% confidence interval and was statistically significant at a p-value less than 0.05 using binary logistic regression analysis. Qualitative data were collected from nine key informants via focused interviews or semi-structured in-depth interviews and analyzed using ATLAS.ti version 7.0.7 software via narratives using the thematic analysis method.

**Result:**

According to the study’s findings, a greater number (43.4%) of the respondents had ineffective collaboration during their professional activities. In the final model of multivariable analysis, unsatisfactory organizational support, poor professional support, and poor interpersonal support were all independently associated with ineffective collaboration. The qualitative findings identified poor communication, a lack of professionalism, and failure to adhere to professional duties as barriers to nurse-physician collaboration.

**Conclusion:**

In this study, nurse-physician collaboration was less than expected; thus, the large number of participants had ineffective collaborations. Potential predictors of decreased effective nurse-physician collaboration included dissatisfaction with organizational support, poor professional support, and poor interpersonal support. This outcome emphasizes the importance of improving nurse-physician collaboration by enhancing organizational, professional, and interpersonal factors to form effective collaborative practice. The qualitative finding supports the quantitative study, which showed ineffective collaboration. The authors recommended that there is a need to empower interprofessional collaboration among nurses and physicians through the creation of a conducive and safe working environment.

## Introduction

Interprofessional collaboration is a process involving mutual and active participation among independent professionals [1]. During which each group of healthcare professionals has the knowledge and skills to provid**e** care, and their interactions are governed by mutually agreed-upon shared norms and visions [1, 2].

Conferring to the WHO framework for IPE and IPC in the report, many health systems and health professionals around the world are disconnected and overwhelmed by meeting unmet health demands [2]. Despite the fact that nursing and medicine work closely together and share a commitment to patient wellbeing, a prevalent type of conflict in hospitals is that between nurses and physicians, which is caused by a lack of daily interprofessional collaborations [2, 3].

Interprofessional collaboration among nurses and physicians in Africa has been ineffective, according to the current available literature; however, the available literature is insufficient to reach the conclusions [3, 4]. Insufficient professional support, poor interpersonal communication, and a lack of attention given to interprofessional collaborations are the factors that contribute to ineffective nurse-physician collaborations [4]. The scarcity of health-care practitioners also has a significant impact on inter-professional collaboration [2, 5]. Furthermore, Sub-Saharan Africa is the most affected region, in which nurse-physician interprofessional collaborations are ineffective [5]. As a result, patient safety, care, and improvement were harmed by poor nurse-physician interprofessional collaborations, which also caused moral discomfort among healthcare workers [4, 5]. Ethiopia, like other sub-Saharan African countries, has really poor interprofessional collaborations among nurses and physicians [6, 7]. However, very few studies are available in the country as a whole [6, 8] as compared to other African countries [8, 9].

In addition, there have been no studies available regarding nurse-physician collaborations that were conducted using a mixed-methods study. Rather few studies are available that were conducted using only qualitative methods [10–12]. As care needs become more complex, it is less likely that a single health care professional will be able to address them alone, emphasizing the importance of collaboration [13]. In a dynamic and complicated care setting, effective collaboration helps to improve patient wellbeing, quality of treatment, and satisfaction [14].

The previous study had a general limitation in that the data collection process was only quantitative, so when combined with the qualitative study findings, it could not provide in-depth details on the problems [11, 15]. This study could be a mixed-methods approach to evaluating the state of inter-professional collaboration and the factors that influence it. This has the potential to generate significant evidence for evidence-based collaborative practice in Ethiopia’s north-west Amhara region to improve inter-professional practice.

There is a need to investigate interprofessional collaborations in teaching and referral hospitals in Ethiopia’s north-west Amhara region to improve professional collaboration, patient satisfaction, and treatment outcomes. It is an actual observed problem in clinical practice that needs to be researched in order to see optimal patient care from health care providers. Therefore, this study tries to assess nurse-physician collaboration and associated factors in teaching and referral hospitals in the north-west Amhara region of Ethiopia in 2022.

### Implications of the study (key messages)

What is already known about this topic? Inter-professional collaboration between nurses and physicians was not well studied in Ethiopia. Why did this study need to be done? The previous study had a general limitation in that the data collection process was only quantitative, thus, it could not provide in-depth details on the problems. What does this study add? This study could be a mixed-methods (concurrent qualitative and quantitative) and could generate significant evidence for evidence-based collaborative practice between nurses and physicians. What impact might this study have on research, practice, or policy? This study provides baseline information to policymakers, health care organizations, and health facility managers for appropriate planning and intervention for interprofessional collaboration of nurses and physicians in the clinical setting.

## Methods

### Study design, setting and period

A concurrent (quantitative, cross-sectional, supported by phenomenological, qualitative) study design was used. This study was conducted in two public specialized hospitals in Bahir Dar city and one specialized hospital in Debretabor town from September to October 2022. Bahir Dar is the capital city of the Amhara region, which is located 575 km from Ethiopia’s capital, Addis Ababa [16]. In Bahir Dar, there are only two specialized public hospitals. Those are FelegeHiwot specialized hospital, where currently, 322 nurses and 94 physicians are permanently employed, and Tibebeghion specialized hospital, where currently 325 nurses and 152 physicians are employed. The other four hospitals in Bahir Dar city are private and primary level hospitals. Debretabor town is located in the capital of the South Gondar Zone, approximately 80 km from Bahir Dar. There is only one specialized public hospital in Debretabor, where currently 202 nurses and 52 physicians are permanently employed [17].

### Source population

All nurses and physicians who were working in Bahir Dar and Debretabor public hospitals.

### Study populations

All randomly selected nurses and physicians who were working in Bahir Dar and Debretabor specialized public hospitals.

### Inclusion criteria

All nurses and physicians working in specialized public hospitals in Bahir Dar and Debretabor were included.

### Exclusion criteria

Nurses and physicians who did not directly involve patients care, such as managers and directors were excluded.

### Sample size, sampling procedure and technique

The actual sample size for the study was determined using a single population proportion formula: {n = [(zα/2)^2^p(1-p)]/d^2^}, n = sample size, zα/2 = 95% confidence level, P = the proportion (6.7%) [18] d = margin of error (0.05). By considering 10% of the non-response rate, the final sample size of the study was 375. Since we considered the covariates (associated factors) from the previous studies using Epi Info statistical calculation with two-sided 95% CI and power 80%, but the sample size that was calculated from those factors was less than 375, we took the sample size calculated from the single population formula (375). All three public specialized hospitals (two in Bahir Dar city and one in Debretabor town) were involved in this study. The sample size for each hospital was proportionally allocated based on the number of nurses and physicians in each hospital. Individuals who fulfilled the inclusion criteria were selected using simple random sampling. Purposive sampling was used to select the study participants for the qualitative assessment portion.

### Data collection tool

The quantitative data were collected using English versions of structured, pre-tested, and self-administered nurse-physician collaborative scale questionnaires [18], which contain 27 items classified into three subscales. Subscales of sharing patient information items, the decision-making process (joint participation in the care), and the relationship between nurse and physician (cooperativeness). Cronbach’s alpha reliability for the previous study was 0.72, [15], and for this study, it was 0.94 from the pretested questioners.

### Data collection procedure

The data were collected for 30 days using both quantitative and qualitative methods. The quantitative data were collected by six BSc nursing professionals and supervised by three supervisors throughout the study. The qualitative data were collected by two MSc nurses supervised by the principal investigator using semi-structured (focused interview) questions. Each participant was audio recorded for 20 min before being converted into words.

### Operational definitions

#### Collaboration

Collective action among professionals that was used to integrate healthcare services for patients [1, 11].

#### Nurse-physician collaboration

the interaction between nurses and physicians, and working for patients and their families to deliver quality of care [3, 11].

#### Effective nurse-physician collaboration

high mean score on the nurse-physician collaboration scale (by taking mean as a reference) ( 3).

#### Organizational support

Health care facilities manager who assists their employed health care professionals as the whole to empower interprofessional collaborations [15].

#### Professional support

A health care workers who has been assisted through professional training or any other method that improves professional activities [13].

#### Inter-personal support

A health care workers individually supported with financial or other personal needs to enhance interprofessional collaborations [15].

### Data management and analysis

For the quantitative part, data were entered, checked, and coded into Epi Data version 4.6 and exported to SPSS version 26 for analysis. Descriptive statistics such as mean, frequency, and percentage were computed and presented by using text, tables, and graphs. Hosmer and Lemshow’s goodness of fit test was used to assess the model’s fitness, yielding a p value of 0.75. A binary logistic regression analysis has been performed to identify factors related to inter-professional collaboration as the dependent variable, with p < 0.05 considered statistically significant. For the qualitative part, all interviews were audio -recorded and then translated into English. The translated word documents were exported into Atlas.ti (version 7.0.7 software) for analysis.

### Data quality control

The questionnaires were pre-tested with 5% of the sample size at Debre Markose Specialized Hospital, but with different participants in a different study area. The principal investigator supervised the data collection processes and checked for completeness. For the qualitative part, data were collected by the principal investigator, at the time of data collection; the interview guide was checked by the principal investigator for completeness and consistency of information.

### Patients and public involvement

All randomly selected nurses and physicians at the TIbebe Gion specialized hospital, FelegeHiwot comprehensive specialized hospital, and Debretaboer specialized hospital were involved.

## Results

### Socio-demographic characteristics of the respondents

Among the 375 questionnaires distributed, 366 were returned, indicating a 97.6% response rate. Among the total participants, 279 (76.2%) nurses and 87 (23.8%) physicians were involved in the study. The mean ages and work experiences of the respondents were 28.14 (SD ± 5.12) and 6.31(SD ± 4.12) years, respectively. Most respondents (83.9%) were between 26 and 30 years old. Almost half of the study participants (47%) had 5 to 10 years of work experience (Table 1).

**Table 1:** Socio-demographic characteristics of the study participants in northwest, Ethiopia 2022 (N = 366)

Sociodemographic characteristics	Respondent	No	%
Sex	Male	220	60.1
	Female	146	39.9
Age	< 25 years	37	10.1
	26−30 years	307	83.9
	31−35 years	26	7.1
Marital status	Single	157	42.9
	Marriage Others (separate, divorce)	203 6	55.5 1.6
Working experience	< 5 years	139	38.0
	5−10 years	172	47.0
	11−15 years	35	9.6
	> 15 years	20	5.5
Responsibility in your working unit	Staff nurse	259	70.8
	Ward coordinator nurse	15	4.1
	Staff doctors	75	20.5
	Case manager and above	10	2.7
	Lecturer	7	1.9
Level of education	Level iv Diploma nurse	8	2.2
	BSc nurse	258	70.5
	MSc nurse and above	13	3.6
	Medical Doctor	75	20.5
	Specialists and above	12	3.3
Working unit/area	Inpatient	156	42.6
	Intensive care units	76	20.8
	Emergency Department	74	20.2
	Out Patient Department	60	16.4

In this qualitative study, 9 participants (5 nurses and 4 physicians) answered in-depth interview (focused interview) questions. Their average age and experience were 28.2 (SD 4.23) and 7.2 (SD 5.12) years. Most participants, 7 (77.8%), were men. The qualitative section of this study was categorized in to three themes and eight themes subthemes (Table 2).

**Table 2:** Themes and subthemes of the qualitative section of the study in northwest, Ethiopia 2022

Themes	Subthemes
The status of collaboration among nurses and physicians	The overall professional collaboration between nurses and physicians
	The experience of professional collaboration in their working unit
Factors hindered collaboration	Lack of supply and medical equipment
	Poor recognition and management system in the hospitals
	Poor communication among professionals
	Professional respect and equality
Factors facilitate collaboration	Develop and implement institutional rules and guidelines
	Professional careers and development

### Nurse − physician collaboration

To identify effective and ineffective collaboration, the mean score for each nurse-physician collaboration measuring item was calculated. One hundred and sixty-six (53.6%) were satisfied with nurse-physician collaboration. Joint participation and nurse-physician relationship, 202 (55.2%) and 211 (57.7%), were satisfied with nurse-physician collaboration, respectively. Overall, there was 56.6% effective inter-professional collaboration between nurses and physicians. In this qualitative study, the overall professional collaboration between nurses and physicians were ineffective, which supports the quantitative study (Table 3).

**Table 3:** Response of participants in interprofessional collaboration among nurse-physicians, North West, Ethiopia 2022

The number of questions was 366.	R	N	R	ST	U	A	Mean
		no	no	no	no	no	
The nurses and the exchange physicians’ opinions to resolve problems related to patient cure or care.	N	13	28	70	88	80	3.62
	P	11	13	16	26	21	
In the event of a disagreement about the future direction of a patient’s care, the nurses and physicians hold discussions to resolve differences in opinion.	N	28	40	90	73	48	3.24
	P	14	17	15	23	18	
The nurses and the physicians discuss whether to continue a certain treatment when that treatment is not having the expected effect.	N	56	77	74	45	27	2.54
	p	30	30	17	8	2	
When a patient is to be discharged from the hospital, the nurses and physician discuss where the patient will continue to be treated and the lifestyle regimen the patient should follow.	N	31	62	71	65	50	3.07
	P	15	24	19	21	8	
When challenged by a difficult patient, the nurses and physicians discuss how to handle the situation.	N	18	36	73	92	60	3.49
	P	5	19	17	22	24	
The nurses and the physicians discuss the problems a patient has.	N	22	25	63	84	85	3.73
	P	4	6	14	31	32	
The nurses and the physicians together consider their proposals about the future direction of patient care.	N	33	64	84	72	26	2.93
	P	13	30	19	15	10	
In the event a patient develops unexpected side effects or complications, the nurses and physicians discuss countermeasures	N	22	43	63	90	61	3.40
	P	15	14	11	28	19	
In the event a patient no longer trusts a staff member, the nurses and physicians try to respond to the patient consistently to resolve the situation.	N	20	34	81	109	35	3.38
	P	10	16	13	27	21	
The future direction of a patient’s care is based on a mutual exchange of opinions between the nurses and the physicians.	N	29	54	70	88	38	3.08
	P	16	28	14	20	9	
The nurses and the physicians seek agreement on signs that a patient can be discharged.	N	29	39	54	81	76	3.50
	P	6	14	18	24	25	
The nurses and the physicians discuss how to prevent medical care accidents.	N	23	37	61	78	80	3.60
	P	3	12	14	32	26	
The nurses and the physicians all know what has been explained to a patient about his/her condition or treatment.	N	17	64	94	70	34	3.01
	P	15	34	15	17	6	
The nurses and the physicians share information to verify the effects of the nurses’ treatment.	N	26	41	67	83	62	3.31
	P	13	26	10	24	14	
The nurses and the physicians have the same understanding of the future direction of the patient’s care	N	38	68	79	62	32	2.80
	P	23	28	20	12	4	
The nurses and the physicians identify the key person in a patient’s life	N	20	29	57	96	77	3.72
	P	1	9	16	30	31	
In the event of a change in the treatment plan, the nurses and physicians have a mutual understanding of the reason for the change	N	26	48	67	67	71	3.35
	P	16	13	11	30	17	
The nurses and the physicians check with each other concerning whether a patient has any signs of side effects or complications.	N	14	44	66	87	68	3.48
	P	12	15	10	36	14	
The nurses and the physicians share information about a patient’s reaction to explanations of his or her disease status and treatment methods.	N	21	50	84	76	48	3.21
	P	16	15	20	27	9	
The nurses, the physicians, and the patient have the same understanding of the patient’s wish for cure and care.	N	43	69	61	65	41	2.90
	P	15	30	19	13	10	
The nurses and the physicians share information about a patient’s level of independence concerning activities of daily living.	N	18	50	84	83	44	3.20
	P	14	22	20	25	6	
The nurses and the physicians can easily talk about topics other than work.	N	42	79	78	57	23	2.70
	P	22	30	17	12	6	
The nurses and the physicians can freely exchange information or opinions about matters related to their work.	N	25	47	82	81	44	3.25
	P	11	17	13	34	12	
The nurses and the physicians express concern for each other when they are exhausted.	N	32	79	72	66	30	2.91
	P	14	21	25	22	5	
The nurses and the physicians help each other.	N	19	61	72	65	62	3.28
	P	9	18	22	29	9	
The nurses and the physicians greet each other every day.	N	15	31	64	91	78	3.72
	P	4	8	15	28	32	
The nurses and the physicians take each other’s schedules into account when making plans to treat a patient together.	N	32	45	85	71	46	3.14
	P	17	18	14	26	12	
Average							3.24

N: B, A: Always, U: Usually, ST: Occasionally, R: Rarely, N: Never, no = number or frequency, N = Nurse, P = physician, R = respondent

### Professional factors

Results on the professional factors show that most respondents (55.2%) report that their collaboration was good for interprofessional collaboration. Two-thirds (66.67%) of respondents state that professionals communicate in a responsive and responsible manner that supports a team approach in this factor-specific item. Furthermore, most participants (227, or 62%) believe that laws and regulations are well-required and well-understood within the groups. Findings from this qualitative study revealed that poor communication among professionals is the most common reason for ineffective collaboration.

### Inter-personal factors

Results on the interpersonal factors showed that 193 (52.7%) respondents reported that their collaboration was good for interprofessional collaboration. Most respondents (269, or 73.5%) report that building mutual trust at the individual and professional levels promotes collaboration, while 266 (72.67%) state that inter-professional collaboration usually requires responsiveness. Most participants in this qualitative study stated that some personal factors also affect the level of nurse-physician collaboration.

### Factors associated with inter-professional collaboration among nurses and physicians

In the final model of multivariable analysis, unsatisfactory organizational support, poor professional support, and poor interpersonal support were all independently associated with ineffective collaboration. Participants who were satisfied with organizational support for inter-professional collaboration were 5.6 times more likely to have effective inter-professional collaboration compared to those who were dissatisfied with organizational support [OAR = 5.622, CI: (3.237, 9.766), p = 0.001]. The odds of effective inter-professional collaboration were 2.4 times higher among participants who had good professional support compared to their counterparts (who were not satisfied with professional support for collaboration) [AOR = 2.433, CI: (1.389, 4.259), P = 0.002]. The odds of effective inter-professional collaboration were approximately twice high among participants who had good interpersonal support compared to those who had poor interpersonal support [AOR = 2.148, CI: (1.237, 3.731)), P = 0.007] (Table 4). In this qualitative study, the main factors that hinder or create barriers to professional collaboration between nurses and physicians are explored.

**Table 4:** Factors associated with inter-professional collaboration among nurses and physicians, North West, Ethiopia 2022

Variable N = 366		Inter-professional collaboration		COR 95% CI	AOR 95% CI	p value
		effective	ineffective			
Sex	Male	114	106	0.613 (0.399, 941) 1:00	0.651 (0.384, 1.104) 1:00	0.111
	Female	93	53			
Organizational support	Unsatisfied	158	36	1: 00	1:00	
	Satisfied	49	123	11.017 (6.75, 17.99) *	5.622 (3.237, 9.766)	0.001*
Professional support	Poor	154	48	1:00	1:00	
	Good	53	111	6.719 (4.240, 10.65) *	2.433 (1.389, 4.259)	0.002*
Inter-personal support	Poor	146	47	1:00	1:00	
	Good	61	112	5.704 (3.625, 8.973) *	2.148 (1.237, 3.731)	0.007*

Note. 1:00: reference, *Significant at p-value < 0.05, CI: Confidence interval, COR: crude odd ratio, AOR: adjusted odd ratio

## Discussion

According to the findings of this study, nearly half (43.4%) of nurses and physicians had ineffective inter-professional collaborations. The findings of this study are consistent with those of a previous study conducted in the Tigray region, which found 45.7% [11] of participants in Tigray and 46.5% of participants in Italy engaged in ineffective interprofessional collaboration [19]. The findings of this study showed that the proportion of ineffective interprofessional collaborations among nurses and physicians (43.4%) were lower than the previous studies conducted in Bahir Dar (58.8%) [10], Addis Ababa (57.3%) [11] and Egypt 61% [20, 21]. This discrepancy could be due to differences in the study period, method, and sample size [11]. For instance, this study is a current (updated) study, used an adequate sample size and mixed methods (multicenter cross-sectional study supported by a phenomenological qualitative analysis). However, the proportion of ineffective nurse-physician collaboration in this study was higher than the studies conducted in Jimma (33.3%) [15], USA (30%) [22], and China (23%) [22]. The difference might be due to differences in study settings and working habits [19]. For instance, this study was conducted in Bahir Dar, Ethiopia, where poor working habits are usually observed [10]. In addition, different study contexts, professional respect, country levels of development, and variations in professional development could be contributing factors to the difference [22]. Most findings in our context therefore suggest that the level of collaboration is low and needs special attention to be improved in order to raise the quality of patient care, improve patient outcomes, and increase patient satisfaction [12].

In this qualitative study, the overall professional collaboration between nurses and physicians was ineffective, which supports the quantitative study. However, no previous qualitative or mixed studies were available to compare to this study.

The results of this study showed that participants who were satisfied with organizational support for collaboration significantly increased their level of inter-professional collaboration. This finding was supported by the qualitative part of this study as well. This result is consistent with previous studies conducted in the Tigray region [12], Kenya [23], Nigeria [24], Iran [25] Canada [26], Norway [27], and USA [28]. As a result, to strengthen interprofessional collaboration among nurses and physicians, organizational support is required. thus achieving quality patient care and improving patient outcomes.

According to the findings of this study, good professional support for inter-professional collaboration is related to increased nurse-physician collaboration. This finding was consistent with previous research conducted in Kenya [23], Nigeria [29], Lebanon [30], Singapore [31] and Iran [25]. Therefore, providing strong evidence-based professional support can improve nurse-physician interprofessional collaborations [32]. This study findings revealed that good inter-personal support for collaboration resulted in more effective inter-professional collaboration. This is consistent with a study conducted in Kenya [23] Canada [26] and USA [28]. The qualitative part of this study also identified that failure to fulfill either nurse or physician roles and responsibilities or both roles and responsibilities, lack of professionalism, poor support and recognition from the hospital and management systems, a lack of medical supplies in their working area, and a lack of professional careers and development were the reasons that could be barriers to collaborations. This is consistent with the study conducted in Dar as Salaam [33], Netherlands [34], and Australia [31].

### Strengths and limitations of the study

The study design was a concurrent (quantitative study supplemented with qualitative) study design, and it recruited an adequate sample size. The findings from this study might be subjected to respondents’ bias. The study also shares the limitations of cross-sectional study design. outcome and exposure complex (the chicken and egg dilemma). However, the authors attempted to address this issue through the use of a concurrent study design.

## Conclusion

In this study, most participants had effective inter-professional collaboration, whereas a considerable number of respondents had ineffective inter-professional collaboration. The results of this study showed that several factors affect collaboration; three factors organizational factors, professional factors, and interpersonal factors were significantly related to collaboration. The qualitative study showed, the overall professional collaboration between nurses and physicians was ineffective, which supports the quantitative findings. The authors recommended that there is a need to empower interprofessional collaboration among nurses and physicians through creating a conducive and safe working environment, providing ongoing training, and holding workshops on the importance of interprofessional collaboration.

The institution could provide supports for professional growth, motivations, and recognitions for professionals, increase professional satisfaction, mutual understanding of roles and enable them to develop a sense of collaborative.

## Data Availability

The data that support the findings of this study are available upon reasonable request from the corresponding authors.

## Abbreviations

ATLAS. I: Archive for Technology, life world And everyday language Text Interpretation
ICP: Interdisciplinary Collaborative Practice
IPC: Inter-Professional Collaboration
IRB: Institutional Review Board
JCAHO: Joint Commission on Accreditation of Health Care Organizations
NPCS: Nurse Physician Collaboration Scale
